# Diagnostic performance of core needle biopsy for nodal recurrences in patients with head and neck squamous cell carcinoma

**DOI:** 10.1038/s41598-022-06102-0

**Published:** 2022-02-07

**Authors:** Ta-Hsuan Lo, Cheng-Ping Wang, Chun-Nan Chen, Tsung-Lin Yang, Pei-Jen Lou, Jenq-Yuh Ko, Yih-Leong Chang, Tseng-Cheng Chen

**Affiliations:** 1 Department of Otolaryngology, National Taiwan University Hospital and National Taiwan University College of Medicine, No.7, Chung-Shan S. Rd., Taipei, 10002 Taiwan, ROC; 2 Department of Otolaryngology, National Taiwan University Hospital Hsin-Chu Biomedical Park Branch, Hsinchu, Taiwan, ROC; 3 Department of Pathology, National Taiwan University Hospital, National Taiwan University Cancer Center and National Taiwan University College of Medicine, Taipei, Taiwan, ROC; 4 Graduate Institute of Pathology, National Taiwan University College of Medicine, Taipei, Taiwan, ROC

**Keywords:** Head and neck cancer, Cancer imaging, Oral cancer

## Abstract

This study investigated the diagnostic accuracy and affecting factors of ultrasound (US)-guided core-needle biopsy (CNB) in patients with treated head and neck squamous cell carcinoma (HNSCC). We retrospectively reviewed patients with treated HNSCC who received US-guided CNB from January 2011 to December 2018 with corresponding imaging. Pathological necrosis and fibrosis of targeted lymph nodes (LNs) were evaluated. We analyzed the correlation between CNB accuracy and clinical and pathological characteristics. In total, 260 patients were included. The overall sensitivity, specificity, positive predictive value (PPV), negative predictive value (NPV), and accuracy of CNB for nodal recurrence were 84.47%, 100%, 100%, 54.67%, and 86.92%, respectively. CNB of fibrotic LNs had significantly worse sensitivity, NPV, and accuracy than that of non-fibrotic LNs. Similarly, CNB of necrotic LNs had significantly worse sensitivity, NPV, and accuracy than non-necrotic LNs. Multivariate regression revealed that fibrotic LN was the only independent factor for a true positive rate, whereas both necrotic LN and fibrotic LN were independent factors for a false negative rate. The diagnostic accuracy of CNB in treated HNSCC patients is affected by LN necrosis and fibrosis. Therefore, CNB results, particularly for necrotic or fibrotic LNs, should be interpreted carefully.

## Introduction

In patients with treated head and neck squamous cell carcinoma (HNSCC), detection of neck nodal recurrence is an important issue. Therefore, imaging techniques like high-resolution ultrasound (US)^1^, computed tomography (CT), magnetic resonance imaging (MRI) and positron-emission tomography (PET)^2–6^ play important roles in the evaluation of suspected neck nodal lesions during follow up. However, imaging studies are only able to provide indirect evidence of the lesion but not direct cytological or pathological evidence to help clinicians differentiate malignant lesions from other conditions affecting the neck, such as deep neck infection, necrosis, or chyle/hematoma accumulation. Therefore, obtaining tissue samples remains the standard requirement for accurate diagnosis of neck nodal recurrence.

In treated HNSCC patients, open biopsy of the neck provides a tissue sample that is ideal for immunohistochemical analysis. However, this procedure usually requires general anesthesia and hospital admission. Previous reports have shown that prior radiation and/or neck dissection can result in significant changes to the histological architecture of the neck, for example, tissue fibrosis or necrosis^7,8^. Therefore, an open surgical biopsy without guidance for the suspicious lesion over treated neck is always difficult and carries risk of injury to the important vessels or nerves. Recently, US-guided lymph node (LN) core-needle biopsy (CNB) has been shown to be as advantageous as open surgical biopsies but without the disadvantages associated with open surgical biopsies^9,10^. It is minimally invasive, safe, simple, and cheap. In addition, it is also a fast outpatient procedure that can be performed under local anesthesia and provides an acceptable cutting tissue core for immunohistochemical study. However, to the best of our knowledge, there have been no comprehensive studies investigating whether changes to the histological architecture in treated neck have any impact on the diagnostic accuracy of cutting tissue core by US-guided CNB. Therefore, in this study, we aim to study the performance of US-guided CNB in treated HNSCC patients. All possible factors, especially the fibrosis and necrosis in targeted LN, will be examined in order to clarify their impact, if any, on the performance of US-guided CNB in detail.

## Results

### Patient demographics

In our study, a total of 260 patients with treated HNSCC who had a suspected neck mass received US-guided CNB were included. Of the 260 patients, 236 (90.77%) were men and 24 (9.23%) were women. Their ages ranged from 30 to 88 years, with a mean age of 57 ± 10 years. The long axis of targeted LN ranged from 0.6 to 5.20 cm, with a mean axis of 2.2 ± 0.8 cm. Totally, there were 41 (15.77%) non-recurrent patients and 219 (84.23%) recurrent patients, including 185 patients confirmed by US-guided CNB and 34 patients confirmed by progressive disease or salvage neck dissection, in our series. The basic clinicopathologic and US characteristics of these recurrent and non-recurrent patients are shown in Table 1. There were significant differences between the patients with non-recurrent and recurrent LN in terms of primary tumor sites (*p* = 0.02), target LN pathological fibrosis (*p* = 0.04) and LN width size (*p* < 0.001).

**Table 1:** Basic, clinicopathologic and ultrasound characteristics of treated head and neck SCC patients with recurrent LN.

Characteristics	Non-recurrent LN (N = 41)	Recurrent LN (N = 219)	*p* value
**Age (years)**			
≧50	29 (70.73%)	173 (79%)	0.31
< 50	12 (29.27%)	46 (21%)	
**Gender**			
Male	38 (92.68%)	198 (90.41%)	0.78*
Female	3 (7.32%)	21 (9.59%)	
**Primary index tumor**			
Oral cancer	15 (36.59%)	121 (55.25%)	0.02*
p16- (< 70%) Oropharyngeal cancer	12 (29.27%)	44 (20.09%)	
p16 + (≧70%) Oropharyngeal cancer	2 (4.88%)	1 (0.46%)	
Hypopharyngeal, Laryngeal cancer	10 (24.39%)	31 (14.16%)	
Others	2 (4.88%)	22 (10.05%)	
**Previous neck management**			
Neck dissection only	9 (21.95%)	64 (29.22%)	0.20
Radiation only	22 (53.66%)	47 (21.46%)	
Neck dissection and Radiation	10 (24.39%)	103 (47.03%)	
No treatment	0	5 (2.28%)	
**Neck recurrence duration**			
Acute (≦6 months)	16 (39.02%)	66 (30.14%)	0.28
Non-Acute (> 6 months)	25 (60.98%)	153 (69.86%)	
**Target LN pathological fibrosis**			
Fibrotic changes+	17 (41.46%)	55 (25.11%)	0.04
Fibrotic changes−	24 (58.54%)	164 (74.89%)	
**Target LN pathological necrosis**			
Necrosis+	8 (19.51%)	34 (15.53%)	0.64
Necrosis−	33 (80.49%)	185 (84.47%)	
**Target LN radiological necrosis**			
Total necrosis	2 (4.88%)	26 (11.87%)	0.29*
Partial necrosis	9 (21.95%)	59 (26.94%)	
No necrosis	30 (73.17%)	134 (61.19%)	
**Targeted LN level in neck**			
Upper (I/II/Va)	25 (60.98%)	150 (68.49%)	0.37
Lower (III/IV/Vb)	16 (39.02%)	69 (31.51%)	
**LN width size**			
≧1.5 cm	21 (51.23%)	179 (81.74%)	< 0.001
< 1.5 cm	20 (48.78%)	40 (18.26%)	
**Ultrasound characteristic**			
Ill-defined	21 (51.22%)	83 (37.90%)	0.12
Heterogenous	20 (48.78%)	89 (40.64%)	0.39
**CNB tissue diagnosis**			
Positive	0	185 (84.47%)	< 0.001
Negative	41 (100%)	34 (15.53%)	

SCC, squamous cell carcinoma; LN, lymph node.

*Using Fisher’s exact test.

### The association between the presence of necrotic/fibrotic LNs and other clinical/pathological characteristics

In our study, there were 72 (27.69%) patients with fibrotic LNs pathologically and 123 (47.31%) patients with necrotic LNs (81 patients with necrotic LNs radiologically, 27 patients with necrotic LNs pathologically, and 15 patients with combined radiologically and pathologically necrotic LNs). Among the 42 patients with necrotic LN pathologically, there were 8 patients with tissue necrosis and 34 patients with tumor necrosis. We attempted to clarify the association between the presence of necrotic/fibrotic LNs and underlying clinical/pathological characteristics of patients with treated HNSCC. Compared with 58 patients aged < 50 years, the odds ratio (OR)s of necrotic/fibrotic LNs in 202 patients aged ≥ 50 years were not significantly different (*p* = 0.47 and 0.72, respectively). Compared to 24 women, the ORs of necrotic/fibrotic LNs in 236 men were not significantly different (*p* = 0.48 and 0.76, respectively). Compared to 201 patients with treated non-oropharyngeal cancer, the ORs of necrotic/fibrotic LNs in 59 patients with treated oropharyngeal cancer were not significantly different (*p* = 0.54 and 0.44, respectively). Compared to 191 patients without prior radiation, the ORs of necrotic LNs in 69 patients with prior radiation was not significantly different (*p* = 0.31). However, the OR of fibrotic LNs was significantly high for the patients with prior radiation (OR 1.91, 95% confidence interval 1.06–3.44, *p* = 0.03). Compared to 178 patients with recurrent duration > 6 months, the ORs of necrotic LNs in 82 patients with recurrent duration ≤ 6 months was not significantly different (*p* = 0.74). However, the OR of fibrotic LNs was significantly high for the patients with recurrent duration ≤ 6 months (OR 1.93, 95% confidence interval 1.10–3.39, *p* = 0.02) Compared to 85 patients with lower neck LN, the ORs of necrotic/fibrotic LNs in 175 patients with upper neck LN were not significantly different (*p* = 0.84 and 0.47, respectively). Finally, compared to 60 patients with LN width size < 1.5 cm, the ORs of necrotic/fibrotic LN in 200 patient with LN width size≧1.5 cm were not significantly different (*p* = 0.88 and 0.86, respectively).

### The sensitivity, specificity, PPV, NPV and accuracy of US-guided CNB tissue sampling method in necrotic and fibrotic LN

**Figure 1:**
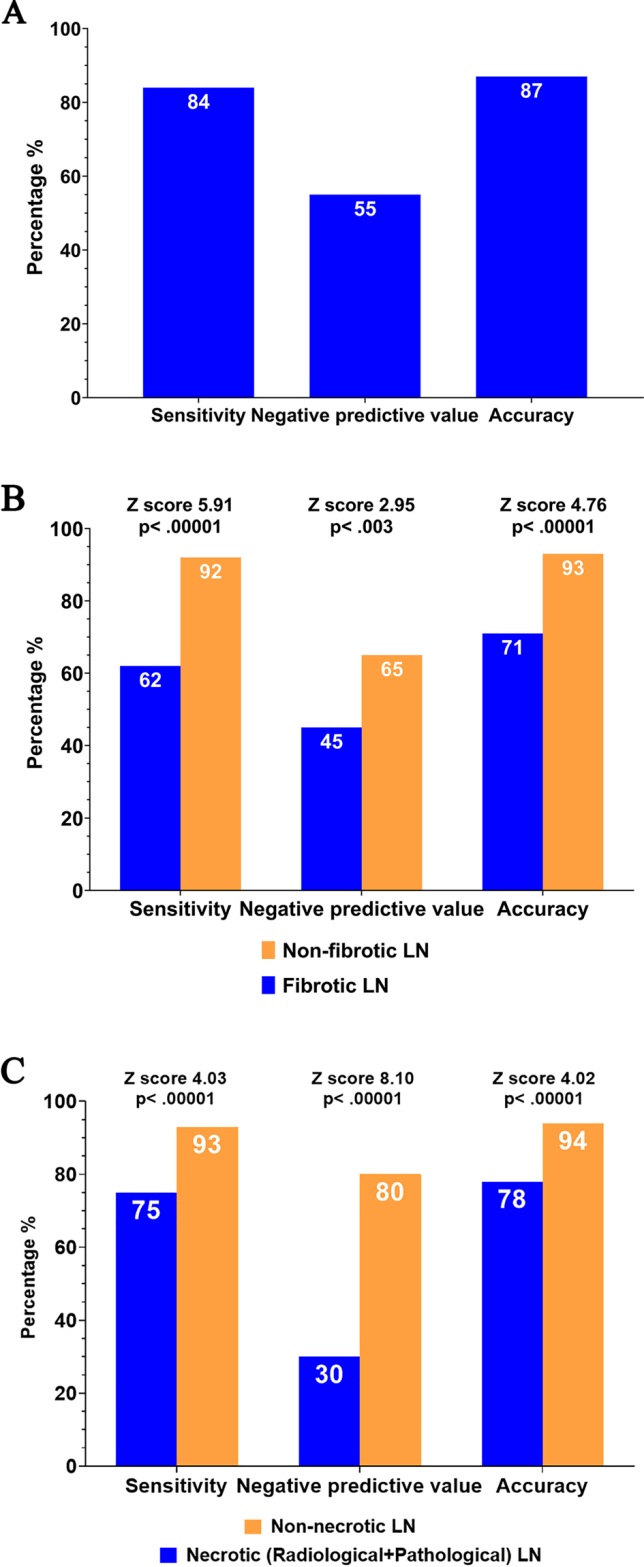
The sensitivity, negative predictive value, and accuracy of (**A**) core-needle biopsy (CNB) in patients with treated head and neck squamous cell carcinoma, overall; (**B**) CNB for the lymph node (LN) with and without fibrotic LN; and (**C**) CNB for the LN with and without necrotic LN.

The overall sensitivity, specificity, PPV, NPV, and accuracy of US-guided CNB in our study were 84.47%, 100%, 100%, 54.67%, and 86.92%, respectively (Fig. 1A). According to the pathological results from CNB, none of the patients had non-diagnostic findings. Compare to the targeted LN without pathological fibrosis, the targeted LN with pathological fibrosis had a significantly worse sensitivity (*p* < 0.00001), NPV (*p* < 0.003) and accuracy (*p* < 0.00001, Fig. 1B). Similarly, compared to the targeted LN without radiological/pathological necrosis, the targeted LN with radiological/pathological necrosis had a significantly worse sensitivity (*p* < 0.00001), NPV (*p* < 0.00001), and accuracy (*p* < 0.00001, Fig. 1C).

**Figure 2:**
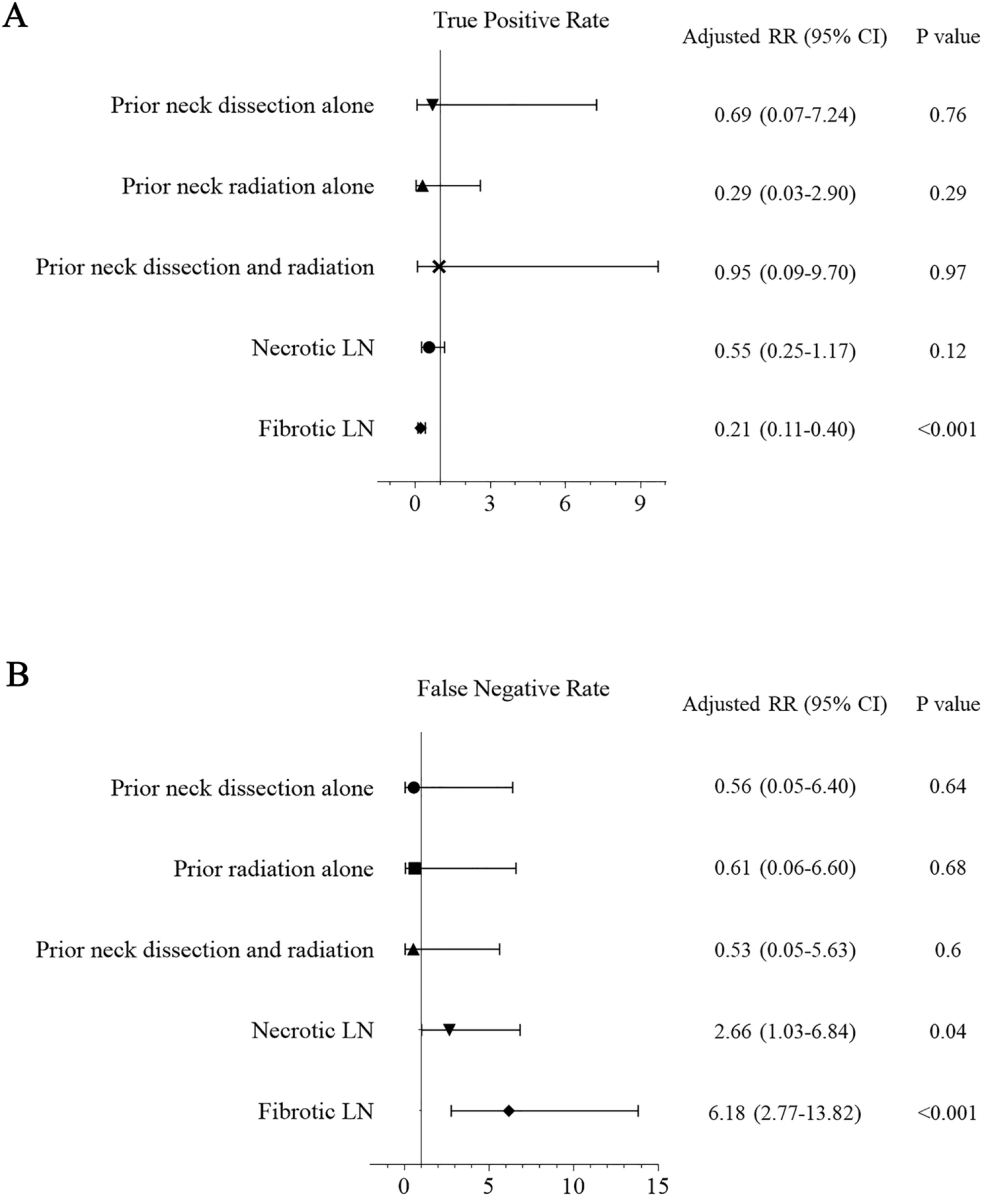
Forest plot of multivariate analysis of possible impacting factors using the logistic regression method in (**A**) true positive rates and (**B**) false negative rates.

Finally, regarding the true positive rates and false negative rates, all of the characteristics between recurrent and non-recurrent patients were further examined by multivariate logistic regression analysis. This analysis revealed that fibrotic LNs (relative risk (RR) 0.21, 95% CI 0.11–0.40, *p* < 0.001, Fig. 2A) was the only independent risk factor for decreased true positive rates. Regarding false negative rates, necrotic LNs (RR 2.66, 95% CI 1.03–6.84, *p* = 0.04) and fibrotic LNs (RR 6.18, 95% CI 2.77–13.82, *p* < 0.001) were independent risk factors for increased false negative rates (Fig. 2B). To summarize, the necrotic LNs and fibrotic LNs had significant impacts on true positive and false negative rates of US-guided CNB.

## Discussion

Previously, US-guided CNB has been reported as an effective, inexpensive, time-efficient, safe, and minimally invasive diagnostic tool for neck nodal disease^11,12^. According to previous reports, the sensitivity, specificity, and accuracy of CNB for neck masses were about 99.7%, 100% and 99.46%, respectively^13,14^. However, data on the diagnostic power of CNB came majorly from neck masses without obvious histological or morphological changes. To the best of our knowledge, no previous work has focused on the possible impact of morphological and histological changes in neck masses on the diagnostic power of CNB. According to our study, several important findings should be emphasized. First, patients with prior neck radiation and recurrent duration ≦ 6 months had significantly high incidence of fibrotic LNs.. Second, necrotic and fibrotic LNs had significant impact on the diagnostic performance of CNB, especially for the true positive and false negative rates.

**Figure 3:**
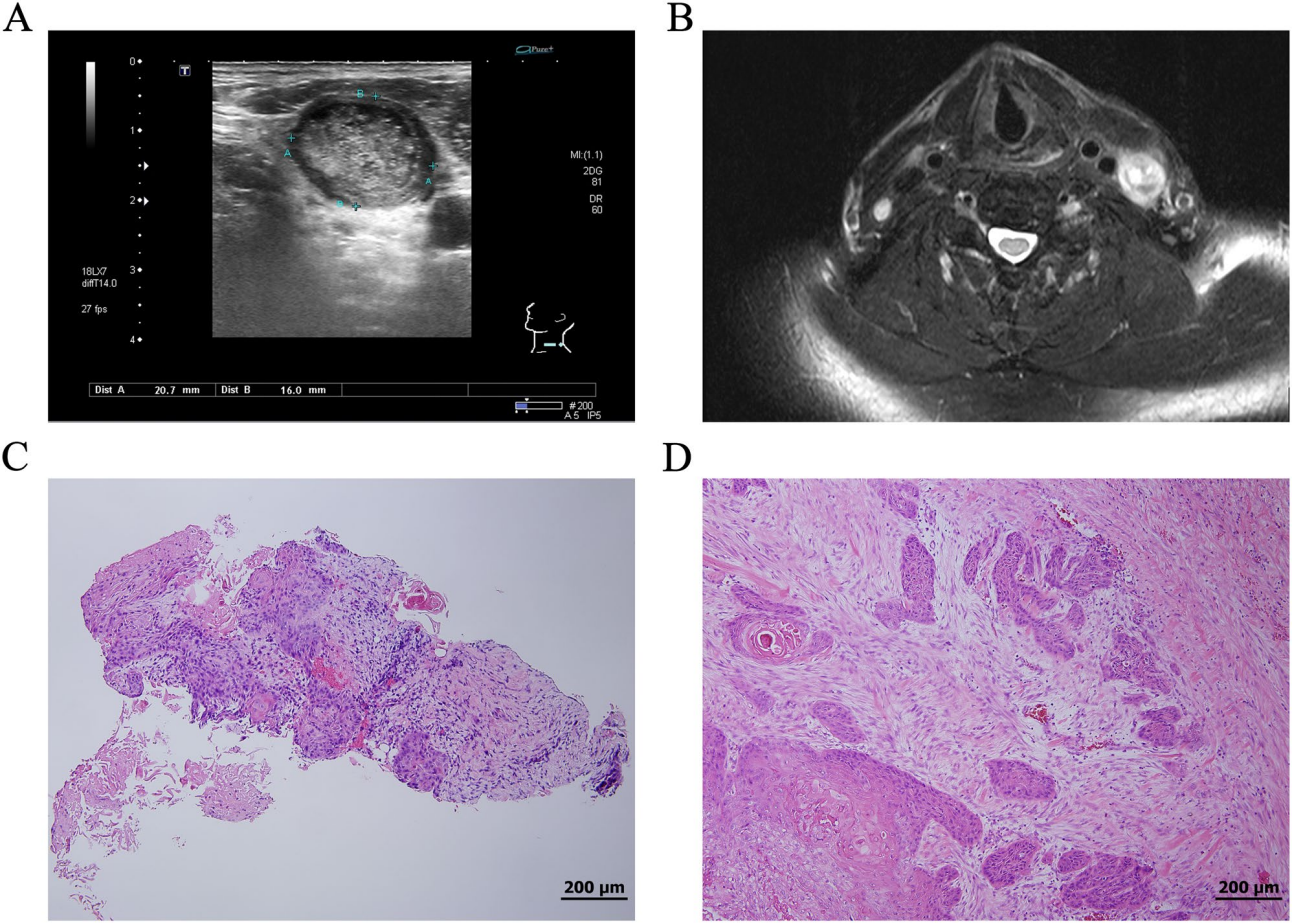
(**A**) Ultrasound image of the treated left tongue cancer with an ipsilateral level IV nodal mass; (**B**) corresponding magnetic resonance imaging showing necrotic changes with a T2 axial view; (**C**) core-needle biopsy (CNB) revealed necrotic tissue with metastatic squamous cell carcinoma (SCC) in nests infiltrated in the fibrotic stroma; and (**D**) tissue from salvage neck dissection revealed metastatic SCC without extranodal extension.

Compared to open surgical biopsy, US-guided CNB had significantly lower sequela and therefore is more accepted by patients^15^, and could be the first choice to obtain tissue samples. However, for patients with HNSCC, it has been reported that the incidence of neck nodal necrosis ranged from 20 to 38%, especially for HPV-related oropharyngeal cancer^16,17^. According to the results of our study, if there is necrotic changes in the target neck mass, the interpretation of CNB findings should be done with caution. In fact, the cutting method used during CNB may only provide partially crushed tissue (Fig. 3) or even no tissue. Previously, it had been reported that nodal necrosis could hinder the accuracy of CNB diagnosis in patients with treated HNSCC^18^. In our study, no clinical characteristics had a significant association with the presence of necrotic lymph nodes in treated HNSCC patients. In our opinion, it should be very important to determine the presence or absence of necrosis in the targeted LN before CNB, especially in patients with treated HNSCC. For LNs with necrotic changes, the clinician should keep in mind that the significantly low NPV of CNB can sometimes cause a dilemma for the diagnosis. In such a situation, excisional biopsy or US-guided FNA should be considered as an alternative diagnostic tool. It had been reported that US-guided FNA had good accuracy in post-radiotherapy pateints^19^. Besides, other functional imaging such as PET may be considered to confirm the negative findings from the CNB.

**Figure 4:**
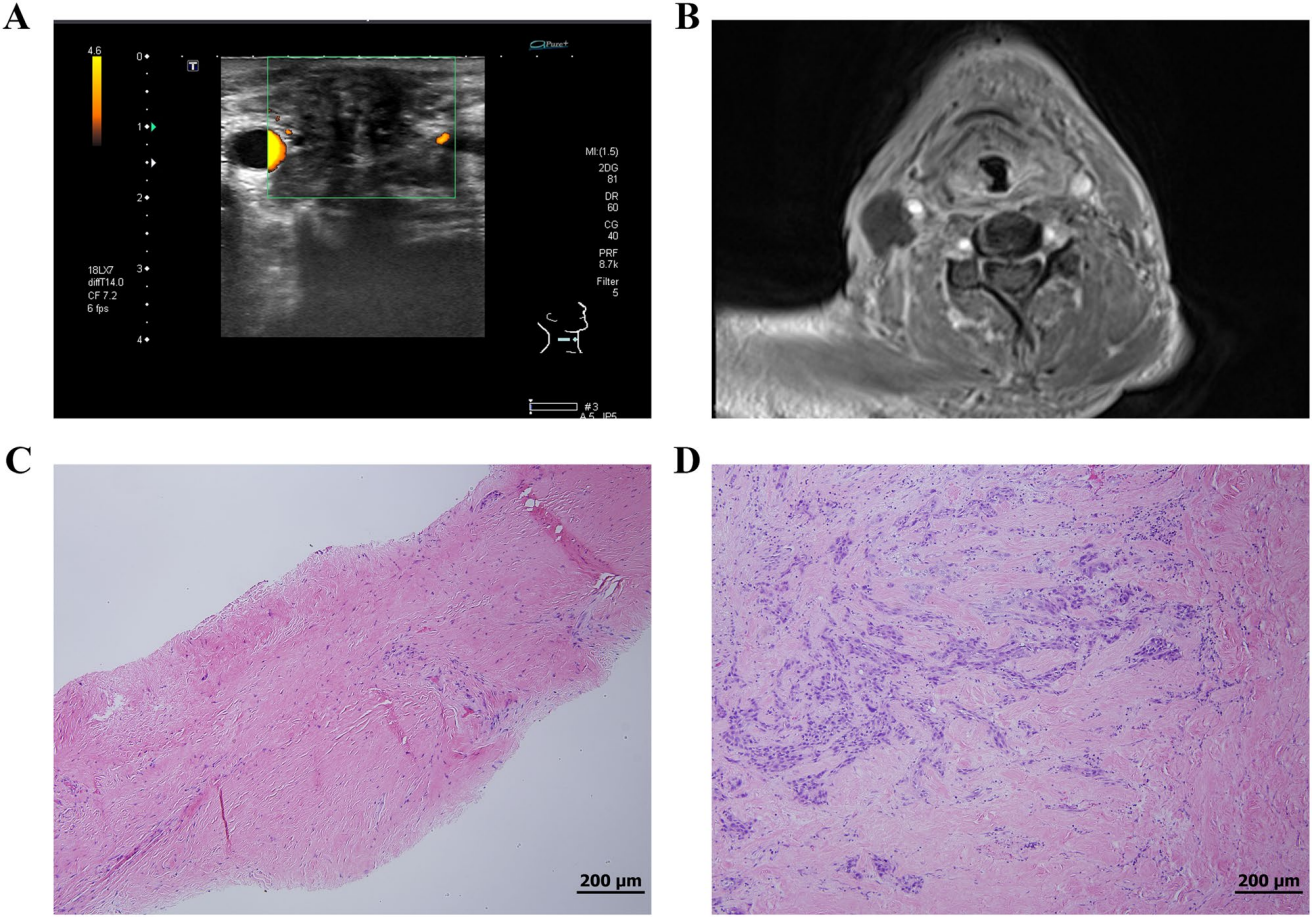
(**A**) Ultrasound picture of treated right tongue cancer with ipsilateral nodal mass; (**B**) corresponding magnetic resonance imaging showing axial view of T1 with gadolinium contrast medium; (**C**) core-needle biopsy revealed fibrotic tissue only; (**D**) Open biopsy revealed fibrotic tissue with metastatic squamous cell carcinoma and focal extracellular keratin deposition.

In our study, in addition to nodal necrosis, pathological fibrosis of the LNs could also hinder the diagnostic accuracy of CNB. Previous neck management, especially radiotherapy, cause an increased production of fibrin. This radiation fibrosis can affect any tissue in the radiation field, including the lymph node^20^. It has been reported that the incidence of neck fibrosis after neck radiation can reach 22%^21^. According to our results, 27% of core tissues from treated LN showed some degree of fibrotic change (Fig. 4). Furthermore, imaging methods such as MRI, CT, or US could not identify the degree of LN fibrotic change before the CNB procedure. Therefore, detection of pathological fibrotic changes majorly depended on the microscopic findings of core tissue obtained from CNB. According to our results, these fibrotic changes significantly hinder the diagnostic accuracy of CNB, especially true positive and false negative rates. Also, the linear one shot procedure of CNB could potentially result in less extensive sampling.

In summary, US-guided CNB still could be the procedure of choice for treated HNSCC patients with a suspected neck mass. However, US-guided CNB could not replace excisional biopsy as the gold standard because its diagnostic accuracy can be significantly affected by necrotic and/or fibrotic LNs. The CNB pathological result should be interpreted with caution if necrosis and/or pathological fibrosis present. If the suspicion of nodal recurrence is high but the findings from CNB are negative, an open biopsy or other functional imaging such as PET should be considered.

There were a few limitations to our study. First, this retrospective study might include various types of bias. Even though multivariant analysis was used to evaluate the factors impacting true positive and false negative rates, there were unavoidable or unnoticed selection biases. Second, in all of the cases, the needle size (18-gauge) used during the CNB procedure was small. Therefore, the effect of different needle size, especially a larger size needle (16-gauge), could not be examined in our study. Third, the number of CNB passes was not available. Therefore, the diagnostic power of CNB may be under- or over-estimated. The strength of this study is that we comprehensively analyzed the association between necrotic/fibrotic changes of the core tissue and the diagnostic accuracy of CNB for treated HNSCC patients. In our opinion, a well-designed prospective randomized trial is warranted in the future to examine the diagnostic power of CNB in patients with treated HNSCC.

## Materials and methods

### Ethical Considerations

The study was approved by the hospital’s Research Ethics Committee (NTUH IRB-201812004RINC) and all methods were performed in accordance with the relevant guidelines and regulations.

### Patient population

We retrospectively reviewed the medical records of neck US from January 2011 to December 2018 at National Taiwan University Hospital. First, all patients with a definite diagnosis of HNSCC were initially included in the study. Further, patients without the history of primary curative treatment before US examination, without US-guided CNB, without corresponding cross-section imaging such as MRI and/or CT at the time point of US examination and without subsequent follow up in our hospital after US examination were excluded from our series. Therefore, all patients included in our series were treated HNSCC patients who underwent neck US-guided CNB with corresponding cross-sectional imaging (MRI or CT) and histological tissue diagnosis by US-guided CNB. Necrotic changes in the aspirated neck mass were determined by corresponding MRI and/or CT imaging before US-guided CNB and were radiologically defined as a central area of low attenuation surrounded by an irregular rim of enhancing tissue^22^. The size of the aspirated neck mass was determined by the long-axis on US measurement. Fibrotic changes in the aspirated neck mass were determined on the basis of the pathologic findings of US-guided CNB tissue block. In our study, all patients in our series received follow-up again 6 months later by routine survey using neck palpation, imaging CT, or MRI to exclude the misdiagnoses of US-guided CNB. The definite diagnoses of LN recurrence were based on the CNB pathologic report or progressive LN during follow up. Furthermore, the TNM stage of HNSCC was determined according to the 2010 criteria of the American Joint Committee on Cancer^23^.

### Procedures of US examination and US-guided CNB

Head and neck US was performed (Toshiba Aplio SSA790 diagnostic US system, Tochigi-ken, Japan or Hitachi HI VISION Avius®, Soto-kanda, Chiyoda-ku, Tokyo, Japan) with a 12-MHz linear array transducer. After obtaining informed consent from the patients, US-guided CNB was carried out. CNB was performed using the free-hand technique with an 18-gauge core-needle (Temno Evolution™ Biopsy Devices, Cardinal Health Inc., Dublin, CA, USA) under local anesthesia, as previously described^1^.

### Statistical analysis

All statistical analyses were performed using the SPSS software package, version 23.0 (SPSS Inc., Chicago, IL, USA). Fisher’s exact tests and Chi-square tests were used to determine differences in the clinical and US features between patients with and those without necrotic LNs. The two-proportion z-test was used to compare the sensitivity, negative predictive value (NPV) and accuracy between the targeted LNs with and without histological fibrosis or radiological necrosis. The primary outcomes were the sensitivity, specificity, positive predictive value (PPV), NPV and accuracy of US-guided CNB to confirm neck nodal recurrence in treated HNSCC patients. The secondary outcomes were difference in sensitivity, NPV and accuracy of US-guided CNB between the targeted LNs with and without histological fibrosis or morphological necrosis. All potential US features were further analyzed using a multivariate logistic regression model. Corresponding *p* values < 0.05 were interpreted as being statistically significant.

## Conclusion

The diagnostic accuracy of CNB in treated HNSCC patients is affected by LN necrosis or fibrosis. Therefore, CNB results, particularly for necrotic/fibrotic LNs, should be interpreted carefully. If the suspicion of nodal recurrence is high but the findings from CNB are negative, an open biopsy or other functional imaging such as PET should be considered, if feasible.
